# Thermal thresholds of the predatory mite *Balaustium hernandezi*

**DOI:** 10.1111/phen.12055

**Published:** 2014-05-14

**Authors:** Megan R Coombs, Jeffrey S Bale

**Affiliations:** School of Biosciences, University of BirminghamEdgbaston, U.K.

**Keywords:** *Balaustium hernandezi*, biological control, chill coma, CT_max_, CT_min_, heat coma

## Abstract

The lower and upper thermal activity thresholds of adult and larval *Balaustium hernandezi* von Heyden (Acari: Erythraeidae) are compared with those of its prey *Tetranychus urticae* Koch (Acari: Tetranychidae). Adult female *B. hernandezi* retain ambulatory function (CT_min_) and movement of appendages (chill coma) at significantly lower temperatures (5.9 and −2.1 °C, respectively) than those of larval *B. hernandezi* (8.1 and −1.7 °C) and *T. urticae* (10.6 and 10.3 °C). There is no significant difference between the temperature at which adult and larval *B. hernandezi* and *T. urticae* cease walking as the temperature is raised (CT_max_) (46.7, 46.3 and 47.3 °C, respectively). However, both life stages of *B. hernandezi* cease movement (heat coma) below the upper locomotory limits of *T. urticae* (46.8, 46.7 and 48.7 °C, respectively). Adult *B. hernandezi* have significantly faster walking speeds than larvae and *T. urticae* across a range of temperatures. The lower thermal activity threshold data indicate that *B. hernandezi* would make an effective biological control agent in temperate climates; however, the extent of the low temperature tolerances of the species suggests the potential to establish in a northern European climate.

## Introduction

Augmentative biological control comprises the application of a natural enemy in areas where abiotic factors prevent prolonged survival and reproduction, and is considered to be an environmentally safe and cost effective method of crop pest management (van Lenteren & Bueno, 2003; van Lenteren, 2011). However, invertebrate augmentative biological control agents must undergo an environmental risk assessment before use in many European Union (EU) countries to prevent the introduction of a species that has the potential to become invasive. There is no existing Europe-wide legislation to regulate the release of invertebrate biocontrol agents, resulting in a difference in the licensing process in different European countries, although a number of countries, including the U.K. and the Netherlands, now operate under a more standardized process (Bale, 2011). To comply with these guidelines, any potential biological control agent must be assessed using appropriate methods to determine the establishment potential and host range (Wapshere, 1974; van Lenteren *et al.*, 2003; Hatherly *et al.*, 2005; van Lenteren & Loomans, 2006). Augmentation is the prevalent form of biological control in Europe, and is used predominantly in glasshouse horticulture. Previous investigations report concentrating on the low winter temperatures preventing the establishment of non-native natural enemies that escape from glasshouses and other protected environments (Tullett *et al.*, 2004; Hatherly *et al.*, 2005; Hughes *et al.*, 2009; Bale, 2011).

As well as preventing establishment, a glasshouse invertebrate biological control agent must be sufficiently effective to warrant application for controlling a pest species (McClay & Balciunas, 2005). Behavioural responses to temperature are an important component in the selection criteria of an invertebrate biocontrol agent because the thermal thresholds of a poikilothermic organism dictate the minimum and maximum temperatures at which activity and life processes can proceed (Huey & Kingsolver, 1989). These parameters provide a preliminary indication of whether a species is likely to survive winter conditions outside of a glasshouse environment, and demonstrate the extent to which the species is able to exert control over a horticultural pest effectively.

The critical thermal minimum (CT_min_) and chill coma are nonlethal values of the lowest temperatures at which an invertebrate can perform motile tasks. The CT_min_ is the lowest temperature at which it is possible for a species to walk in a coordinated manner (Cowles & Bogert, 1944). The organism can retain use of its appendages at temperatures below the CT_min_ but, on entering chill coma, all movement ceases (Mellanby, 1939). Providing that the exposure temperature increases, invertebrates are usually able to regain use of limbs (chill coma recovery) and eventually to walk in a coordinated manner (activity recovery). However, if the ambient temperature remains below the chill coma temperature for an extended period, the invertebrate may be killed by the low temperature exposure (Mellanby, 1939).

Similarly, upper thermal thresholds are the values of the highest temperatures resulting in an invertebrate losing the ability to walk in a coordinated manner (CT_max_) and use of the appendages (heat coma) (Mellanby, 1939; Cowles & Bogert, 1944). Previous studies of a variety of invertebrates report that heat coma is effectively also the upper lethal limit (Hazell *et al.*, 2010; Hughes *et al.*, 2010a,b[Bibr b25],[Bibr b26]). Although recovery from CT_max_ is recorded in *Atta sexdens rubropilosa* (Hymenoptera: Formicidae), there is no significant difference found between the temperatures at which the predatory mites *Phytoseiulus macropilis* and *Phytoseiulus persimilis* (Acari: Phytoseiidae) and the herbivorous mite *Tetranychus urticae* Koch (Acari: Tetranychidae) encounter CT_max_ and heat coma (Ribiero *et al.*, 2012; Coombs & Bale, 2013).

The present study compares the thermal activity thresholds of *Balaustium hernandezi* von Heyden (Acari: Erythraeidae), a candidate glasshouse biological control agent, with that of its target the phytophagous crop pest *T. urticae*. *Balaustium hernandezi* is a recently described species originating from Spain, and initially found in glasshouses maintained by Biobest N.V., Westerlo, Belgium in 2008, with a rearing population being established by the company in 2010 (Mąkol *et al.*, 2012). Invertebrate biological control agents are applied annually to at least 20 000 ha of glasshouses in Spain (Pilkington *et al.*, 2010). As a native species, *B. hernandezi* could be applied to either glasshouse or open field environments in Europe as an effective spider mite predator, without contravening any biological control regulations. However, to comply with the environmental risk assessment requirements of countries outside the native range, the thermal activity thresholds of *B. hernandezi* should be characterized as an indicator of dispersal ability. Ideally, the activity thresholds and walking speeds of the predator should broadly reflect those of its prey. The present study tests the hypothesis that the thermal thresholds and motility of *B. hernandezi* are at least equivalent to those of the target prey.

## Materials and methods

### Rearing of Balaustium hernandezi and Tetranychus urticae

The initial populations of *B. hernandezi* were supplied by Biobest NV (Belgium). The predators were cultured in a sealed container (12 × 18 × 6 cm). The rearing boxes were half filled with soil and sprayed daily with water to provide a water source for mites. The boxes were kept in quarantine at 25 °C under an LD 18 : 6 h photocycle to reflect commercial rearing regimes (Mąkol *et al.*, 2012). Adults obtained from the commercial source were allowed 2 weeks under rearing conditions before use in the experiments. This allowed time to mate and acclimate to the rearing regime. The boxes were then retained under rearing conditions, and the soil was sprayed daily until larval hatch.

*Tetranychus urticae* were reared as a food source for the predators on French bean plants *Phaseolus vulgaris* L. (Fabaceae) in quarantine at 22 °C and under an LD 18 : 6 h photocycle. Mites were transferred from damaged plants to fresh *P. vulgaris* on a weekly basis to maintain a large population. Infested *P. vulgaris* leaves were added daily to the *B. hernandezi* rearing boxes to ensure that predators had a continuous supply of prey.

### Experimental system

The experimental system followed that developed by Hazell *et al.* (2008), which has previously been used to assess the thermal activity thresholds of a variety of invertebrate species (Hazell *et al.*, 2010; Hughes *et al.*, 2010a,b[Bibr b25],[Bibr b26]). Mites were placed in an arena (diameter of 25 mm, depth of 7.5 mm) in an aluminium block that allowed the passage of cooled or heated fluids from a connected alcohol bath (Haake Phoenix 11 P2; Thermo Electron Corp., Germany). The arena was covered with a clear plastic Petri dish and the walls of the arena were coated with Fluon (Blades Biological, U.K.) to prevent the escape of the mites. A type K thermocouple connected to a thermometer (Tecpel Advanced Digital Thermometer DTM-315; Heatmiser Ltd, U.K.) was inserted into the arena wall, which recorded the temperature throughout the experiment.

CT_min_, chill coma and recovery experiments were conducted in a controlled environment room at 10 °C. CT_max_ and heat coma experiments were conducted in a similar room at 23 °C. Although the aluminium block serves as a conductor for the temperature of the alcohol flowing within it, the temperature within the arena varies spatially as a result of the external temperature (Piyaphongkul, 2013). To negate the difference in temperatures experienced by the mites according to their location in the arena, a calibration was undertaken. Three replicates of five mites were attached to type K thermocouples using Oecotak (Oecos, U.K.) and placed in different locations in the arena. The temperature of the arena used for lower thermal thresholds was reduced from 25 to −12 °C, and the arena used for upper thermal thresholds was increased from 25 to 60 °C. The temperatures of each of the five mites were recorded at regular intervals. The results were compared with the temperature recorded by the thermocouple in the wall of the arena using linear regression. All experimental results were then calibrated using the results of the linear regression.

The thermal activity thresholds of adult female and larval *B. hernandezi* and adult female *T. urticae* were recorded for retrospective analysis using an Infinity 1 digital camera (Lumenera, Canada) with a × 10 macro lens (MLH-10X; Computar, CBC Corp., New York, New York). Videos were recorded using studio capture and analyzed using studio player and studio measure (Studio86Designs, U.K.).

### CT_min_ and chill coma

A sample of six mites was transferred into the arena and the temperature was reduced at a rate of 0.2 °C min^−1^ from 25 to −4 °C because preliminary experiments demonstrated that 100% of *B. hernandezi* entered chill coma before this temperature was reached. CT_min_ was recorded as the temperature at which each individual made a final coordinated ambulatory movement, and chill coma as the temperature at which the final twitch of an appendage was made. Thirty individuals were monitored for entry into CT_min_ and chill coma.

### Chill coma and activity recovery

Thirty fresh individuals were used to measure chill coma and activity recovery. Samples of six mites were transferred into the arena, and the temperature was reduced from 25 to −4 °C at a rate of 0.5 °C min^−1^. This rate was used to minimize any hardening responses from the mites (Hazell *et al.*, 2008). The arena was held at −4 °C for 10 min to ensure that all individuals had entered chill coma, and then returned back to 25 °C at a rate of 0.2 °C min^−1^. As the temperature increased, chill coma recovery was recorded as the temperature of the earliest twitch of an appendage, and activity recovery as the temperature at which the mite resumed coordinated locomotion.

### CT_max_ and heat coma

Thirty individuals of each species were observed for entry into CT_max_ and heat coma. Samples of six mites were transferred into the arena, and the temperature was increased from 25 to 60 °C at a rate of 0.2 °C min^−1^. CT_max_ was measured as the temperature at which each individual made a final coordinated ambulation, and heat coma as the temperature at which the final twitch of an appendage was made. Because entry into heat coma effectively comprised the upper lethal temperature, it was not possible to record recovery from heat coma.

### Walking speed

Thirty individuals were observed to determine the mean walking speed at a range of temperatures. Samples of three mites were transferred into the arena. The temperature of the arena was raised from 25 to 30 °C, and individuals were recorded for 10 min. The temperature of the arena was then lowered at 5 °C intervals to 0 °C at 0.5 °C min^−1^, holding the mites at each progressively lower exposure temperatures for 10 min. The first 5 min of each exposure were allocated to negate any lag in temperature between the arena and the mites; therefore, only the final 5 min of each exposure was analyzed. The distance that each mite travelled over the 5 min was measured, from which the mean walking speed could be calculated.

### Statistical analysis

All statistical analyses were carried out using minitab, version 15 (Minitab Ltd, U.K.). *Tetranychus urticae* data were sourced from Coombs & Bale (2013).

The critical thermal minimum and maximum, entry into chill and heat coma, and resumption of activity were initially analyzed using distribution ID plots, which confirmed the most appropriate distribution for use in further analyses of the results. In all cases, the Weibull distribution was considered the best fitting and therefore most suitable, and is a commonly utilized distribution in the analyses of life data (Cohen, 1965). Upon determining that the data were normally distributed, parametric distribution analyses could be performed using the Weibull distribution. These analyses identify differences in both shape and scale of the data between each species. Significant differences within the data were confirmed using one-way analysis of variance and Tukey's honestly significant difference post-hoc tests.

The walking speed data were not normally distributed; accordingly, the nonparametric Scheirer–Ray–Hare extension of the Kruskal-Wallis test was determined to be the most appropriate for analyzing the data. *P* < 0.05 was considered statistically significant.

## Results

### CT_min_ and chill coma

There was a significant difference between the temperatures at which each species entered CT_min_ (*P* < 0.001; χ^2^ = 58.58; d.f. = 4). *Balaustium hernandezi* adults and larvae were able to retain ambulatory function to lower mean temperatures (5.9 and 8.1 °C, respectively) than *T. urticae* (10.6 °C). The predatory mites encountered CT_min_ at a more narrow range of temperatures than the prey species (Table 1).

**Table 1:** Mean ± SE and range (in brackets) of temperatures (°C) at which adult and larval *Balaustium hernandezi* and adult *Tetranychus urticae* experienced CT_min_, chill coma, chill coma recovery, activity recovery, CT_max_ and heat coma

Species (cohort)	CT_min_	Chill coma	Chill coma recovery	Activity recovery	CT_max_	Heat coma
*Balaustium hernandezi* (adult ♀)	5.9 ± 0.3	−2.1 ± 0.1	0.4 ± 0.4	13.2 ± 0.3	46.7 ± 0.1	46.8 ± 0.1
	(4.6–10.2)^a^	(−2.6 to −1.3)^d^	(−2.7–12.0)^e^	(11.1–15.6)^f^	(45.3–47.9)^g^	(46.0–47.9)^g^
*Balaustium hernandezi* (larvae)	8.1 ± 0.6	−1.7 ± 0.2	−0.5 ± 0.2	13.0 ± 0.2	46.3 ± 0.4	46.7 ± 0.3
	(5.3–14.2)^b^	(−3.4 to −0.2)^d,e^	(−3.5–1.2)^e^	(10.9–17.3)^f^	(43.0–50.9)^g^	(44.4–50.9)^g^
*Tetranychus urticae* (adult ♀)	10.6 ± 0.5	10.3 ± 0.5	12.2 ± 0.6	12.8 ± 0.6	47.3 ± 0.9	48.7 ± 0.7
	(5.3–16.2)^c^	(4.7–15.7)^c^	(8.4–20.4)^f^	(8.4–20.4)^f^	(39.4–54.9)^g^	(43.8–55.2)^h^

Means followed by different superscript lowercase letters are significantly different (Tukey's honestly significant difference, *P* < 0.05).

Source of *T. urticae* data: Coombs & Bale (2013).

Mean chill coma temperature was also significantly lower in *B. hernandezi* adults and larvae compared with *T. urticae* (−2.1, −1.7 and 10.3 °C, respectively) (*P* < 0.001; χ^2^ = 377.67; d.f. = 4). The range of temperatures at which both life stages of *B. hernandezi* experienced chill coma was narrow (1.3 and 3.2 °C, respectively) compared with a difference of 11.0 °C in *T. urticae* (Table 1). There was a significant difference between the mean temperatures at which *B. hernandezi* adults (*P* < 0.001; *F*_1,58_ = 954.25) and larvae (*P* < 0.001; *F*_1,58_ = 214.0) ceased ambulation and twitching of appendages; however, there was no significant difference between the temperatures at which *T. urticae* demonstrated these behaviours (*P* = 0.610; *F*_1,58_ = 0.26).

### Chill coma and activity recovery

Adult and larval *B. hernandezi* recovered use of limbs at a lower temperature than *T. urticae* (0.4, −0.4 and 12.2 °C, respectively). There was a difference in the shape and scale of the distribution of chill coma recovery temperatures between the two species (*P* < 0.001; χ^2^ = 453.74; d.f. = 4). The range of temperatures at which adult *B. hernandezi* recovered from chill coma spanned 14.7 °C, which was much greater than the range of temperatures where the species entered chill coma (Table 1). In comparison, larval *B. hernandezi* recovered across a range spanning 4.7 °C, similar to the range over which this life stage experienced chill coma (3.6 °C).

There was a significant difference between the temperatures at which *T. urticae* entered CT_min_ and chill coma, and the temperatures at which the species recovered the use of its limbs and ambulation (*P* = 0.006; *F*_3,116_ = 4.39). By contrast, adult *B. hernandezi* demonstrated all four lower thermal threshold behaviours at significantly different temperatures (*P* < 0.001; *F*_3,104_ = 402.29) (Table 1). Activity recovery occurred at a higher temperature than chill coma recovery (*P* < 0.001; *F*_1,46_ = 410.15) and was not significantly different from the temperature at which *T. urticae* recovered ambulation (13.2 and 12.8 °C, respectively) (*P* = 0.651; *F*_1,46_ = 0.21).

### CT_max_ and heat coma

There was no significant difference between the temperatures at which adult or larval *B. hernandezi* and *T. urticae* entered CT_max_ (46.7, 46.2 and 47.2 °C, respectively) (*P* = 0.422; *F*_2,87_ = 0.87). Although both species ceased movement at similar temperatures, adult *B. hernandezi* entered CT_max_ over the narrowest range of temperatures compared with larvae and *T. urticae* (Table 1). Larval *B. hernandezi* survived the effects of increasing temperature beyond the limits of the adults (50.9 and 47.9 °C, respectively).

Although there was no significant difference between the temperatures at which *T. urticae* ceased ambulation and twitching of its appendages (*P* = 0.251; *F*_1,58_ = 1.35), the herbivore retained control of its limbs to higher temperatures than *B. hernandezi* (*P* < 0.001; χ^2^ = 372.51; d.f = 4). There was no significant difference between CT_max_ and heat coma thresholds in adult *B. hernandezi* (*P* = 0.231; *F*_1,58_ = 1.46) or larval (*P* = 0.395; F_1,58_ = 0.73). The range of temperature over which adult and larval *B. hernandezi* experienced heat coma was narrower than that of *T. urticae* (Table 1).

### Walking speed

**Fig. 1:**
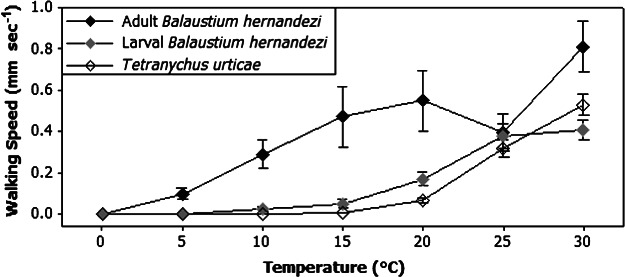
Mean ± SE walking speed (mm s^−1^) at different temperature intervals of adult and larval stages of the predatory mite *Balaustium hernandezi* compared with that of the target prey species, the two spotted spider mite *Tetranychus urticae*. Source of *T. urticae* data: Coombs & Bale (2013).

There was a significant difference between the walking speeds of *B. hernandezi* and *T. urticae* (*P* < 0.001; *H* = 18.80; d.f. = 2). Adult *B. hernandezi* were able to move significantly faster than larvae and *T. urticae* at all temperature intervals between 10 and 30 °C, except for 25 °C (Fig. 1). Although adult *B. hernandezi* appeared to move at a higher speed at 5 °C compared with larvae and *T. urticae*, examination of the Bonferroni 95% confidence intervals showed no significant difference. There was no significant difference between the walking speed of larval *B. hernandezi* and *T. urticae* at any temperature (*P* = 0.697; *H* = 0.151; d.f. = 1).

## Discussion

Invertebrate behaviour is dictated by temperature and, as such, the study of thermal thresholds has particular importance with respect to the assessment of the suitability of a species for biological control. Thermal activity thresholds demonstrate the limits of the ambulation ability of a poikilothermic organism, which will have a direct influence on life processes such as predator avoidance, oviposition, foraging and prey consumption (Crist & MacMahon, 1991; Gotoh *et al.*, 2004; Ahnesjö & Forsman, 2006; Berger *et al.*, 2008). An augmentative biological control agent intended for use in a temperate region, such as *B. hernandezi*, should preferably retain locomotor function at temperatures rendering the target prey immobile, and match or exceed the speed of prey movement.

As shown in the present study, *B. hernandezi* maintains coordinated movement at temperatures below that of its prey, and therefore has the capacity to forage when environmental conditions render *T. urticae* immobile. The species is proposed as a candidate biological control agent because it is observed to predate whitefly, spider mite and thrips (Mąkol *et al.*, 2012). However, there are wider safety implications for use of the species outside its native distribution because the mite can retain use of its limbs at subzero temperatures, and recovers from chill coma at 0.4 °C. Because the CT_min_ and chill coma temperatures are usually higher than the lower lethal limit of the species, the data indicate that *B. hernandezi* may be able to survive colder conditions (Mellanby, 1939). If *B. hernandezi* displays a robust cold tolerance strategy, can survive a typical northern European winter and can reproduce successfully, long-term establishment is possible. Maintaining ambulation at low temperatures also maximizes the opportunity that mites have to seek refugia (in places such as tree bark, pedicels of fruit or leaf litter) at the onset of more adverse conditions (Veerman, 1992). The refugia offer a more stable microclimate, protecting the individual from the adverse conditions of winter, and thus increase the chance of survival to the next spring (Danks, 2002). The combination of these factors may render the species unsuitable for use as an augmentative biological control agent as a result of the chance of glasshouse escapee survival and establishment (Hatherly *et al.*, 2005; M. Coombs and J. Bale, unpublished observations).

The temperature range of 7–12 °C is noted to slow the movement of the related species *Balaustium* sp. nr. *putmani*, which aggregate at lower temperatures (Yoder *et al.*, 2010). This behaviour is hypothesized to comprise a mechanism for alleviating water stress, and is observed in other acarine species such as *Dermatophagoides farinae* (Acari: Pyroglyphidae) and *Alaskozetes antarcticus* (Acari: Cryptostigmata) (Block & Convey, 1995; Glass *et al.*, 1998; Benoit *et al.*, 2008). The ensuing cluster reduces the exposed surface area for each individual, and the internal area of the aggregation will have a higher relative humidity, reducing the risk of desiccation (Lockwood & Story, 1986). Because the mites in the present study are at very low densities during the thermal threshold and walking speed experiments, no aggregation behaviour is recorded. However, clustering behaviour in glasshouse escapees may aid establishment of the species.

*Balaustium hernandezi* is able to resist the deleterious effects of increasing temperature to 47 °C, demonstrating a considerable tolerance of the effects of heating. The CT_max_ of many tropical arthropods is lower, for example: 34.9 °C in the brown planthopper *Nilaparvata lugens* (Homoptera: Delphacidae) (Piyaphongkul *et al.*, 2012); 40.0 °C in the Argentine ant *Linepithema humile* (Hymenoptera: Formicidae) (Jumbam *et al.*, 2008); and 42.6 °C in the predatory mite *P. macropilis* (Coombs & Bale, 2013). A higher CT_max_ is found to correlate with hotter native environments, albeit very weakly (Addo-Bediako *et al.*, 2000; Deutsch *et al.*, 2008). However, *B. hernandezi* is found at lower latitudes, and has a Mediterranean native distribution (Mąkol *et al.*, 2012). The xeric nature of the origins of *B. hernandezi* may account for the increased tolerance of high temperatures. A comparison between several populations of the Hawaiian frut fly species *Drosophila mimica* (Diptera: Drosophilidae) demonstrates that individuals with dry distributions are more resistant to desiccation than their mesic conspecifics (Eckstrand & Richardson, 1980).

Several species within the genus *Balaustium* are reported as being fast moving (Cadogan & Laing, 1977; Yoder *et al.*, 2006, 2007, 2010). The walking speed trials in the present study demonstrate that adult *B. hernandezi* can move significantly more quickly than larval *B. hernandezi* and *T. urticae*. Speed of movement increases up to 30 °C, although adult walking speed slows to mirror that of larvae and *T. urticae* at 25 °C. Because both life stages of *B. hernandezi* match or exceed the walking speed of the target pest, this predatory species should make an effective biological control agent against *T. urticae*.

The majority of previous non-native biocontrol agents released into glasshouses across northern Europe originate from outside of the EU; for example, *P. persimilis* and *Amblyseius swirskii* (Acari: Phytoseiidae) are sourced from Chile and Israel, respectively (Cock *et al.*, 2010). Signatories of the ‘Convention on Biological Diversity’ are committed to Access and Benefit Sharing (ABS), whereby countries hold sovereign rights to their own genetic resources. Twenty of the largest commercial producers of biological control agents are located within the EU, and will be affected by the implementation of ABS: ABS increases the cost of investigating exotic species, and may prevent it entirely (van Lenteren *et al.*, 2011). Biological control companies located within the EU region may in future focus more attention on native EU species such as *B. hernandezi*. However, if the full European distribution of the species is unknown, it is impossible to know in which countries *B. hernandezi* is non-native and would therefore require regulation. Further investigations of the thermal biology of the species will indicate whether it is likely to survive a typical northern European winter, and therefore whether it is suitable for release in countries outside of its native distribution.

The data reported in the present study demonstrate that *B. hernandezi* has the potential to be an effective biological control agent in Spain because its thermal behavioural thresholds mirror or exceed those of the target prey *T. urticae*. However, because *B. hernandezi* can maintain motility below 10 °C and retain some movement at subzero temperatures, further investigations are required to identify the full potential for establishment in northern European countries without current native populations. Investigation of the thermal thresholds indicates a significant potential for dispersal of escapees from glasshouses during winter conditions.
